# Drug Resistance in Multiple Myeloma: Tumor‐Intrinsic Mechanisms and the Bone Marrow Microenvironment

**DOI:** 10.1002/cbf.70286

**Published:** 2026-08-21

**Authors:** Yixuan Chen, Wenming Huang, Mingxuan Tang, Xiaotao Wang

**Affiliations:** ^1^ Laboratory of Hematology Guilin Medical University Guilin China; ^2^ Department of Hematology The First Affiliated Hospital of Guilin Medical University Guilin China

**Keywords:** bone marrow microenvironment, cell communication, drug resistance, immunotherapy, multiple myeloma, signal transduction

## Abstract

Multiple myeloma (MM) is a malignant plasma cell disorder, and despite substantial improvements in prognosis achieved through chemotherapy, immunotherapy, and autologous stem cell transplantation, most patients ultimately develop relapsed or refractory disease. Drug resistance (DR) is increasingly recognized as a dynamically evolving ecosystem shaped by tumor‐intrinsic plasticity and continuous remodeling of the bone marrow microenvironment (BMME), rather than as a single molecular lesion. This review summarizes the major mechanisms of resistance across key drug classes, including alterations in drug targets and signaling nodes, rewiring of apoptotic, proteostatic, and metabolic circuits, and BMME‐dependent protection. We highlight how these processes converge on a limited set of survival hubs and collectively raise the apoptotic threshold under therapeutic pressure. The key to overcoming DR is to conceptualize it as an evolving ecosystem, thereby enabling rational, mechanism‐based combination and sequencing strategies that may prolong progression‐free survival and move MM closer to a functional cure.

## Significance

- This review integrates key mechanisms of drug resistance across major therapeutic classes in multiple myeloma and highlights how bone marrow microenvironmental cues and tumor‐intrinsic bypass signaling converge to sustain disease persistence.
- By framing resistance as a coordinated, multi‐layered process rather than isolated pathways, this work discusses how a unified conceptual framework can inform rational combination and treatment‐sequencing strategies and facilitate the identification of actionable targets.
- Collectively, these insights provide updated perspectives and practical directions for future mechanistic investigations and translational research aimed at overcoming therapeutic resistance in multiple myeloma.

**Abbreviations**
ABC: ATP‐binding cassette
ADCC: antibody‐dependent cell‐mediated cytotoxicity
ADCP: antibody‐dependent cellular phagocytosis
APRIL: A proliferation‐inducing ligand
ATRA: All‐trans retinoic acid
BAFF: B‐cell activating factor
BCL‐2: B‐cell lymphoma 2
BCMA: B cell maturation antigen
BH4: tetrahydrobiopterin
BMECs: bone marrow endothelial cells
BMME: bone marrow microenvironment
BMSCs: bone marrow stromal cells
BRD4: bromodomain containing protein 4
BTZ: bortezomib
CAM‐DR: cell‐adhesion mediated drug resistance
CAR‐T: chimeric antigen receptor T
CCL3: C‐C chemokine ligand 3
CCR1: C‐C chemokine receptor 1
CDC: complement‐dependent cytotoxicity
CFZ: carfilzomib
CRBN: cereblon
Cul4: cullin 4
CXCL12: C‐X‐C motif chemokine ligand 12
CXCR4: C‐X‐C chemokine receptor type 4
c‐MYC: cellular myelocytomatosis oncogene
DCs: dendritic cells
DDB1: DNA damage‐binding protein 1
DDI2: DNA‐damage inducible 1 homolog 2
DEX: dexamethasone
DR: drug resistance
DUBs: deubiquitinating enzymes
E1: ubiquitin‐activating enzyme 1
EAT‐2: Ewing's sarcoma‐associated transcript 2
ECs: endothelial cells
ER: endoplasmic reticulum
ERK: extracellular signal‐regulated kinase
EVs: extracellular vesicles
E‐selectin: endothelial selectin
GCs: glucocorticoids
GRE: glucocorticoid response element
GRs: glucocorticoid receptors
HA: hyaluronic acid
HSP: heat shock protein
IGF‐1: insulin‐like growth factor‐1
IGF‐1R: insulin‐like growth factor 1 receptor
IKZFs: Ikaros family zinc finger proteins
IL‐6: interleukin‐6
IMiDs: immunomodulatory drugs
IRE1α: inositol‐requiring enzyme 1 α
IRF4: interferon regulatory factor 4
JAK: Janus kinase
JAM‐A: junctional adhesion molecule A
mAbs: monoclonal antibodies
MAC: membrane attack complex
MCL‐1: myeloid cell leukemia 1
MCs: myeloid cells
MDSCs: myeloid‐derived suppressor cells
MM: multiple myeloma
NEDD8: neural precursor cell‐expressed developmentally downregulated protein 8
NEU: neutrophil
NF‐κB: nuclear factor‐κB
NK: natural killer
NOTCH3: neurogenic locus notch homolog protein 3
Nrf1: NF‐E2‐Related Factor 1
OCs: osteoclasts
PI3K: phosphoinositide 3‐Kinase
PIs: proteasome Inhibitors
PSMA3: proteasome 20S Subunit α 3
PSMB5: proteasome 20S Subunit β 5
P‐gp: P‐glycoprotein
ROC1: regulator of cullin 1
Rpn13: regulatory particle non‐ATPase 13
RPS3: ribosomal protein S3
RRMM: relapsed or refractory multiple myeloma
SEs: super‐enhancers
SFM‐DR: soluble factor–mediated drug resistance
SIRPα: signal regulatory protein α
SLAMF7: signaling lymphocytic activation molecule family member 7
STAT3: signal transducer and activator of transcription 3
SUMOylation: small ubiquitin‐like modifier modification
TACI: transmembrane activator and CAML interactor
TAMs: tumor‐associated macrophages
TNTs: tunneling nanotubes
Treg: regulatory T cell
TRIP13: thyroid hormone receptor interactor 13
Ub: ubiquitin
UPR: unfolded protein response
VCAM‐1: vascular cell adhesion molecule 1
VLA‐4: very late antigen 4
XBP1: X‐box binding protein 1
XPO1: exportin 1

## 1 Introduction

Multiple myeloma (MM) is a malignant hematologic neoplasm arising from clonal plasma cells in the bone marrow and is characterized by abnormal proliferation of these cells and excessive production of monoclonal immunoglobulins or free light chains. This dysregulated process leads to myeloma‐defining events, including osteolytic bone disease, renal impairment, anemia, and hypercalcemia [1, 2]. Epidemiologic studies indicate that MM accounts for approximately 1% of all malignancies and about 10% of hematologic malignancies [3]. Based on data from the Global Burden of Disease project and population‐based cancer registries, the incidence and mortality burden of MM rose steadily from 1990 to 2021 [4]. Overall, with global aging and regional disparities in diagnosis and treatment, MM is shifting from a rare disorder to a chronic relapsing malignancy of growing public health importance. At the pathogenetic level, the development of MM is initiated by primary immunoglobulin heavy‐chain gene translocations or hyperdiploidy, followed by the accumulation of diverse secondary genetic events and clonal evolution, which collectively shape its marked biological heterogeneity [2]. Various cellular components within the bone marrow microenvironment (BMME) provide a protective niche that not only drives disease progression but also markedly attenuates the efficacy of anti‐myeloma agents [5].

### 1.1 Tumor‐Intrinsic Survival Pathways in MM

The pathophysiological characteristics and therapeutic sensitivity of MM are largely shaped by complex tumor‐intrinsic genetic, epigenetic, and metabolic abnormalities [1, 2]. In addition to early immunoglobulin heavy‐chain translocations and hyperdiploid alterations, secondary genetic events collectively drive clonal evolution and disease progression [2]. Biologically, malignant plasma cells are characterized by highly developed protein synthesis and secretory machinery, rendering them uniquely dependent on the ubiquitin–proteasome system to eliminate misfolded proteins and alleviate proteotoxic stress [3, 6]. When proteostasis is disrupted, cells activate the unfolded protein response (UPR) as an adaptive mechanism to buffer cellular stress. However, MM cells can frequently rewire these homeostatic cascades and constitutively activate core pro‐survival signaling pathways [7, 8]. In addition, tumor‐intrinsic drug resistance is reflected by a systematic elevation of the apoptotic threshold, mediated in part by upregulation of anti‐apoptotic B‐cell lymphoma 2 (BCL‐2) family proteins such as myeloid cell leukemia 1 (MCL‐1) and BCL‐2, as well as by mutations in specific drug‐target subunits [9]. Emerging evidence further indicates that intrinsic metabolic reprogramming, disruption of imbalance in mitochondrial membrane potential homeostasis, and aberrant intracellular nucleic acid‐sensing responses collectively enable MM cells to evade programmed cell death induced by antitumor drugs [8, 10].

### 1.2 The Ecosystem of the BMME

The BMME is not merely an anatomical background but an active and dynamically remodeled ecosystem that provides a critical protective niche for the long‐term survival and therapeutic escape of MM cells [5, 11]. At the cellular level, the BMME comprises specialized stromal and immune lineages. Bone marrow stromal cells (BMSCs) and osteoclasts, through direct physical contact and paracrine signaling networks, stimulate tumor growth and contribute to the development of osteolytic bone disease [11]. Concurrently, the immune landscape within the microenvironment is reshaped toward an immunosuppressive state, characterized by enrichment of myeloid‐derived suppressor cells, M2‐polarized tumor‐associated macrophages (TAMs), and regulatory T‐cells, collectively impairing NK cell‐ and T‐cell‐mediated immune surveillance [10]. At the non‐cellular level, an extracellular matrix‐rich network physically anchors MM cells and triggers multiple intracellular survival signals. In parallel, a dense paracrine network composed of soluble cytokines and extracellular vesicles (EVs) continuously activates bypass signaling pathways within tumor cells, ultimately establishing a microenvironmental sanctuary that protects MM cells from cytotoxicity [1, 5].

## 2 Existing Therapies and Resistance Mechanisms in Multiple Myeloma

International guidelines recommend proteasome inhibitors (PIs), immunomodulatory drugs (IMiDs), and glucocorticoids (GCs), often in combination with autologous stem cell transplantation, as first‐line therapy, thereby markedly improving median survival compared with conventional chemotherapy [3, 12]. In recent years, immunotherapeutic approaches such as monoclonal antibodies (mAbs), bispecific antibodies, and chimeric antigen receptor T (CAR‐T) cell therapy have advanced rapidly, achieving unprecedented depth of response in patients with relapsed or refractory multiple myeloma (RRMM), and are being progressively incorporated into earlier lines of therapy, thereby further reshaping the treatment landscape of MM [13] (Table 1). However, regardless of the depth of the initial response, the vast majority of patients ultimately relapse, and a subset further progresses to disease that is refractory to even multiple classes of agents for which the prognosis is dismal [1]. Current evidence indicates that drug resistance (DR) in MM does not result from a single pathway, but rather from the interaction between tumor‐intrinsic factors (such as alterations in drug targets, dysregulation of apoptotic programs, enhanced DNA repair, and metabolic reprogramming) and the extrinsic protection provided by the BMME (including cell adhesion, cytokine networks, immune escape, and metabolic coupling) [5, 30]. Against this background, integrating resistance mechanisms and BMME‐driven networks is urgently needed to optimize combination and sequencing strategies, uncover actionable targets, and improve long‐term outcomes in MM.

**Table 1:** FDA‐approved medications for MM.

Drug class	Representative agents	Primary target	Landmark trial and NCT
Immunomodulatory drugs	Thalidomide [14]	Cereblon	THAL‐MM‐003 (NCT00057564)
	Lenalidomide [15]		MM‐009 (NCT00056160)
	Pomalidomide [16]		MM‐003 (NCT01311687)
Proteasome inhibitors	Bortezomib [17]	The 20S proteasome	APEX (NCT00048230)
	Carfilzomib [18]		ASPIRE (NCT01080391)
	Ixazomib [19]		TOURMALINE‐MM1 (NCT01564537)
Glucocorticoids	Dexamethasone [20]	Glucocorticoid Receptors	ECOG E4A03 (NCT00098475)
Monoclonal antibodies	Daratumumab [21]	CD38	POLLUX (NCT02076009)
	Isatuximab [22]	CD38	ICARIA‐MM (NCT02990338)
	Elotuzumab [23]	Signaling lymphocytic activation molecule family member 7	ELOQUENT‐2 (NCT01239797)
Antibody–drug conjugates	Belantamab mafodotin [24]	B cell maturation antigen	DREAMM‐7 (NCT04246047)
Selective inhibitors of nuclear export	Selinexor [25]	Exportin 1	STORM (NCT02336815)
Chimeric antigen receptor T‐cell therapy	Idecabtagene vicleucel [26]	B cell maturation antigen	KarMMa (NCT03361748)
	Ciltacabtagene autoleucel [27]		CARTITUDE‐1 (NCT03548207)
Bispecific T‐cell engagers	Teclistamab [28]	B cell maturation antigen × CD3	MajesTEC‐1 (NCT03145181; NCT04557098)
	Talquetamab [29]	G protein‐coupled receptor class C group 5 member D × CD3	MonumenTAL‐1 (NCT03399799; NCT04634552)

### 2.1 Immunomodulatory Drugs

The primary molecular target of IMiDs is cereblon (CRBN), the substrate receptor of the Cullin 4‐RING E3 ubiquitin ligase complex. By binding to CRBN, IMiDs alter the substrate specificity of this complex [31]. Upon IMiD binding, CRBN recruits the key plasma cell transcription factors IKZF1 and IKZF3, members of the Ikaros family zinc finger proteins (IKZFs), leading to their ubiquitination and subsequent proteasomal degradation [31, 32]. Depletion of IKZFs reduces the expression of interferon regulatory factor 4 (IRF4) and its downstream cellular myelocytomatosis oncogene (c‐MYC) axis, thereby suppressing survival and proliferation signaling in MM cells and exerting direct anti‐myeloma effects [31]. IMiDs also enhance antitumor responses in vivo through immunomodulatory effects, including augmentation of T‐cell and natural killer (NK) cell activity and suppression of pro‐tumorigenic cytokines, thereby complementing their direct cytotoxicity against MM cells [33].

One of the major mechanisms underlying clinical resistance involves alterations in CRBN, including mutations, copy number loss, or enhancer methylation‐associated downregulation, which weakens the binding of IMiDs to CRBN or impairs CRBN function, thereby reducing their cytotoxic effect on MM cells [34, 35]. Beyond changes in CRBN, upregulation of competitive substrates or binding partners that interact with IKZF proteins can partially block IKZF degradation, thereby attenuating the effects of IMiDs [36]. Ubiquitination is a chain reaction; loss or inactivation of the chain‐elongating E2 prevents efficient polyubiquitin assembly even when IMiDs recruit substrates to CRBN, limiting degradation and blunting IMiD activity. This liability provides a rationale for synthetic‐lethal combinations that reinforce the CRBN ubiquitination axis [37].

**Figure 1:**
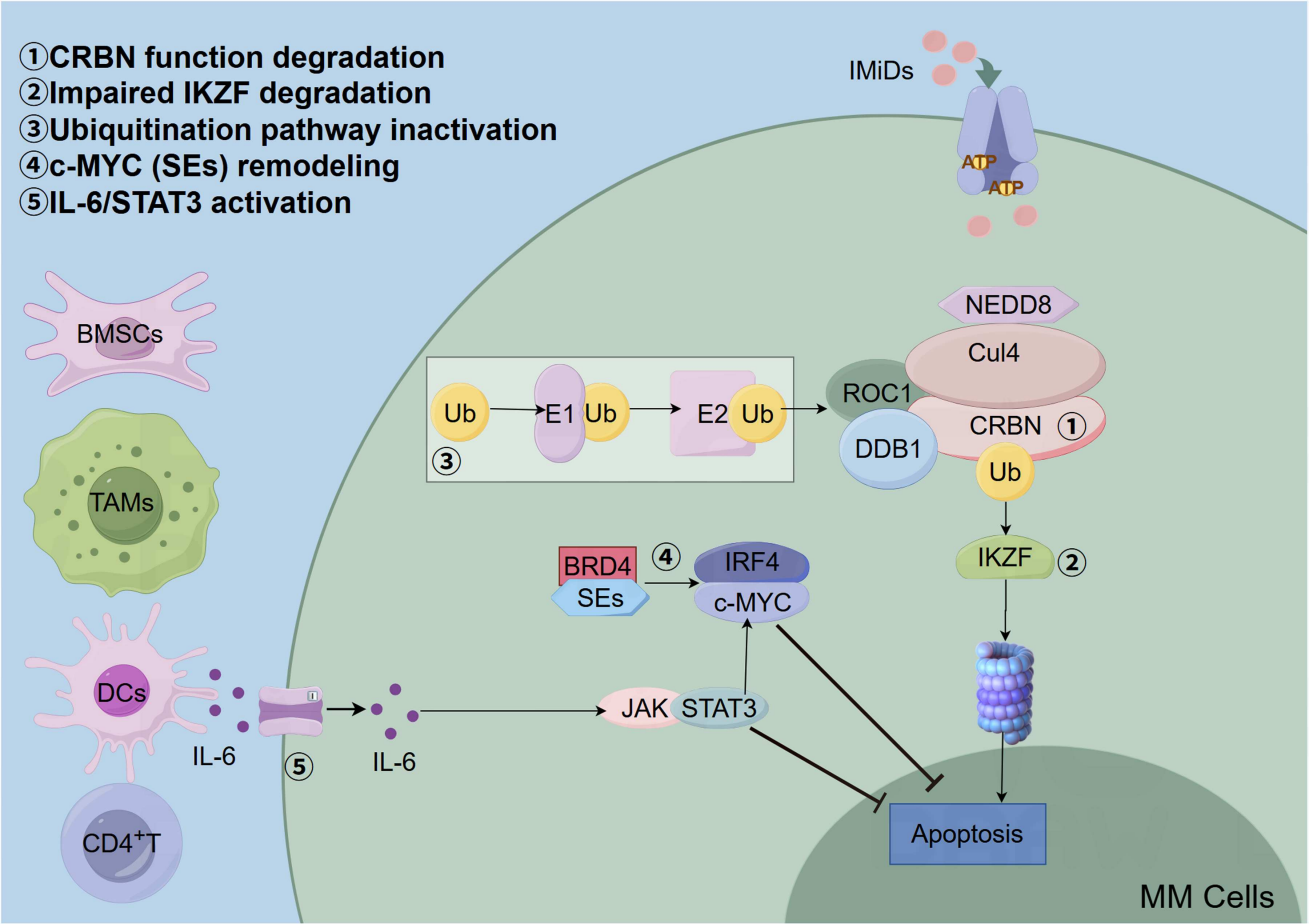
Mechanisms of IMiDs action and resistance in MM.

Beyond CRBN‐related resistance, remodeling of the IRF4–MYC axis and super‐enhancers (SEs) play a pivotal role in the mechanisms of MM resistance. Studies have shown that, even when IKZF proteins are degraded, a subset of tumor cells can sustain high IRF4 and MYC expression through SEs reconfiguration and transcriptional heterogeneity [7, 38]. This observation suggests that combining bromodomain containing protein 4 (BRD4) inhibitors with IMiDs may be an effective strategy to overcome this mode of resistance [38, 39]. Persistent activation of the interleukin‐6 (IL‐6)/signal transducer and activator of transcription 3 (STAT3) signaling pathway is a major driver of drug resistance in MM, prompting the proposed combination of STAT3 inhibitors with IMiDs to restore sensitivity [7, 40]. Beyond direct tumor‐cell inhibition, this approach seeks to interrupt a self‐reinforcing BMME cytokine loop (Figure 1). Mechanistically, IL‐6 supports MM survival by inducing miR‐21, whose upregulation closely tracks STAT3 activation [41]. This combined approach, which simultaneously targets BMME cues and intracellular signaling bypass pathways, offers a novel conceptual framework for the treatment of MM.

Canonical Action: IMiDs bind to CRBN, the substrate receptor of the Cullin 4‐RING E3 ubiquitin ligase complex, altering its surface conformation to recruit IKZF1 and IKZF3. This leads to their polyubiquitination and subsequent proteasomal degradation, which downregulates the downstream IRF4–c‐MYC transcriptional axis, thereby suppressing survival signaling and triggering apoptosis in MM cells. This direct cytotoxicity is complemented by microenvironmental immunomodulation.

Mechanisms of Resistance: (1) CRBN loss or dysfunction: Genomic mutations, copy number loss, or enhancer methylation‐associated downregulation weaken the binding affinity of IMiDs to CRBN or impair E3 ligase complex integrity, thereby blocking the initiation of the drug response at the target level. (2) Impaired IKZFs degradation: The upregulation of competitive cellular substrates or binding partners that interact with IKZF proteins physically shields them from CRBN‐mediated recruitment, protecting these transcription factors from drug‐induced destruction. (3) Defective ubiquitination: The loss or inactivation of the specific chain‐elongating E2 enzyme halts the assembly of polyubiquitin chains on recruited IKZFs, preventing their proteasomal degradation even when the drug successfully binds to the CRBN complex. (4) Maintenance of IRF4–c‐MYC expression via SEs: Driven by transcriptional heterogeneity and super‐enhancer reconfiguration, BRD4 stabilizes SE hubs to maintain high, autonomous expression of the c‐MYC and IRF4 oncogenic axis, creating a CRBN‐independent survival branch that bypasses upstream IKZF depletion. (5) IL‐6/STAT3 signaling activation: Persistent stimulation by BMME‐derived soluble IL‐6 activates the intracellular JAK/STAT3 signaling cascade and upregulates downstream miR‐21. This microenvironmental cue establishes a powerful pro‐survival bypass loop that counteracts pro‐apoptotic death signals. The figure was created by the authors using Figdraw (https://www.figdraw.com/) and does not reproduce or adapt any previously published material.

Abbreviations: IMiDs (Immunomodulatory Drugs), MM (Multiple Myeloma), CRBN (Cereblon), IKZF (Ikaros Family Zinc Finger proteins), IRF4 (Interferon Regulatory Factor 4), c‐MYC (Cellular Myelocytomatosis Oncogene), SEs (Super‐enhancers), BMME (Bone Marrow Microenvironment), Cul4 (Cullin 4), NEDD8 (Neural Precursor Cell‐expressed Developmentally Downregulated Protein 8), ROC1 (Regulator of Cullin 1), DDB1 (DNA Damage‐binding Protein 1), Ub (Ubiquitin), E1 (Ubiquitin‐activating Enzyme 1), BRD4 (Bromodomain Containing Protein 4), BMSCs (Bone Marrow Stromal Cells), TAMs (Tumor‐Associated Macrophages), DCs (Dendritic Cells), IL‐6 (Interleukin‐6), JAK (Janus Kinase), STAT3 (Signal Transducer and Activator of Transcription 3).

### 2.2 Proteasome Inhibitors

MM cells bear an exceptionally high immunoglobulin synthesis burden and are therefore highly dependent on the ubiquitin‐proteasome system to clear misfolded proteins and maintain proteostasis [42]. The 26S proteasome is composed of a 19S regulatory particle and a 20S core particle, within which the proteasome 20S subunit β5 (PSMB5) serves as the direct target of PIs [43]. By inhibiting β5 proteolysis, PIs cause misfolded protein accumulation and UPR activation; when endoplasmic reticulum (ER) stress overwhelms the cellular adaptive capacity, the UPR switches from cytoprotective to pro‐apoptotic signaling, driving the antitumor activity of PIs [42]. In addition to UPR induction, PIs enhance cytotoxicity through several other mechanisms, including activation of the mitochondrial apoptotic pathway and disruption of tumor‐BMME adhesion [44].

In vitro experiments and case studies have shown that PSMB5 mutations can confer cross‐resistance to different classes of PIs [9], and alterations in other subunits such as Proteasome 20S Subunit α 3 (PSMA3) have also been reported [45]. Guided by mutation profiles, selection of carfilzomib (CFZ) over ixazomib, or combination of PIs with a β2‐selective inhibitor has shown efficacy in overcoming acquired resistance and stroma‐mediated protection [9]. Pharmacologic targeting of the 19S proteasome‐associated ubiquitin receptor such as regulatory particle non‐ATPase 13 (Rpn13) has been shown to overcome bortezomib (BTZ) resistance [46]. Nevertheless, targeting Rpn13 has shown synergy with IMiDs in cell‐based studies. Despite ongoing debate regarding the binding sites and selectivity of early compounds, converging evidence supports this approach, and current efforts are focused on optimizing tool molecules for improved selectivity and drug‐like properties [47].

Studies have shown that tetrahydrobiopterin (BH4) promotes BTZ resistance by upregulating deubiquitinating enzymes (DUBs). Inhibition of the BH4–DUBs axis blocks substrate deubiquitination and is effective against BTZ‐resistant clones in vitro and in animal models, and acts synergistically with IMiDs [48]. Sec. 61 delivers nascent polypeptides into the ER. By blocking the ER translocation of newly synthesized proteins, Sec. 61‐translocon inhibitors induce terminal UPR and impose intense proteotoxic stress in MM cells already resistant to PIs and IMiDs [49, 50]. Upon PIs exposure, cells activate NF‐E2–related factor 1, which after processing by DNA‐damage inducible 1 homolog 2 and related factors, translocates to the nucleus and upregulates proteasome assembly chaperone 1 together with multiple 26S proteasome subunits, thereby promoting proteasome reassembly and counteracting PI effects [51]. X‐box binding protein 1 is a regulator of the UPR, and mutations in this protein or downregulation of its signaling pathway can partially impede plasma cell differentiation, reduce ER stress, and have been linked to resistance to PIs [52].

**Figure 2:**
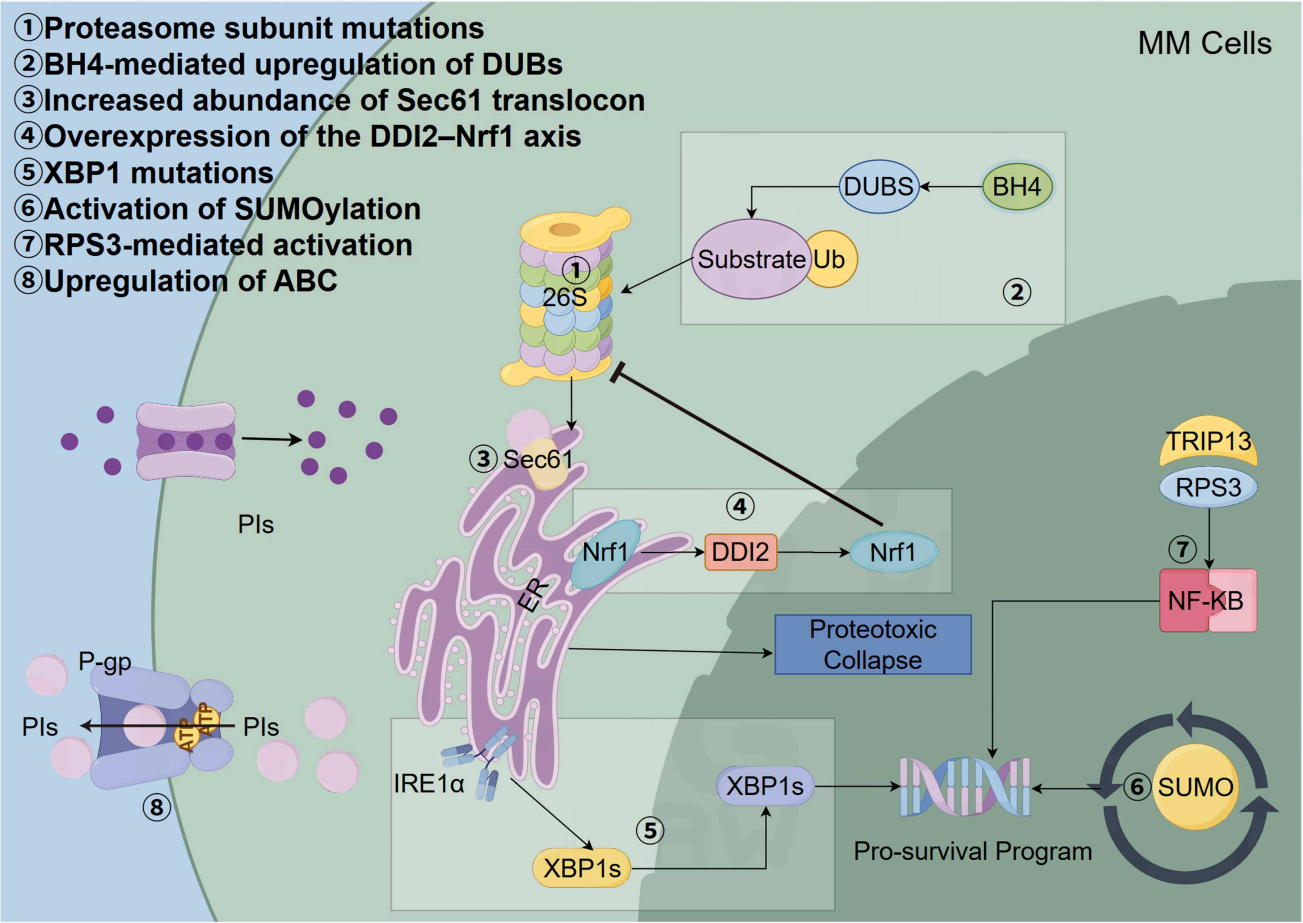
Mechanisms of PIs action and resistance in MM.

Small ubiquitin‐like modifier (SUMO) conjugation, or SUMOylation, confers cellular protection under conditions of DNA damage and proteotoxic stress. Accordingly, combined use of SUMO E1 activating enzyme inhibitors with CFZ can simultaneously impair repair capacity and protein‐folding capacity [53]. During PIs‐induced UPR and proteotoxic apoptosis, HSP90 sustains proteostasis while stabilizing pro‐proliferative and antiapoptotic client proteins, enabling stress buffering and resistance. Accordingly, HSP70 inhibitors are under active clinical development, primarily as components of rational combination strategies to enhance anti‐myeloma efficacy [54]. In addition, ribosomal protein S3 (RPS3) has been identified as a key determinant of DR. Targeting RPS3‐interacting partner thyroid hormone receptor interactor 13 suppresses the nuclear factor‐κB (NF‐κB) signaling pathway and reverses resistance, an effect that has been validated in animal models [55]. Overexpression of ATP‐binding cassette (ABC) transporters promotes drug efflux, reducing intracellular drug exposure and facilitating the emergence of therapeutic resistance. Overexpression of P‐glycoprotein is closely associated with resistance in MM cells to multiple anticancer drugs [6, 56] (Figure 2).

Canonical Action: PIs directly target and inhibit the PSMB5 of the 20S core particle within the 26S proteasome. This blockade prevents the clearance of misfolded proteins, leading to their toxic accumulation and the subsequent activation of the UPR. When intense ER stress overwhelms the cellular adaptive capacity, the UPR switches from a cytoprotective to a pro‐apoptotic signal, driving the activation of the mitochondrial apoptotic pathway, suppressing DNA repair, and disrupting tumor‐BMME adhesion to eliminate MM cells.

Mechanisms of Resistance: (1) Proteasome subunit mutations and adaptive rebalancing: Point mutations in PSMB5 disrupt the drug‐binding pocket to confer cross‐resistance, while alterations occur in PSMA3 and PSMB6 subunits. (2) BH4‐mediated upregulation of DUBs: BH4 promotes resistance by upregulating DUBs, thereby enhancing substrate deubiquitination and reducing the accumulation of ubiquitinated proteins. (3) Increased Sec. 61‐dependent ER translocation: The Sec. 61 translocon complex continuously delivers nascent polypeptides into the ER to maintain high protein load. (4) DDI2–Nrf1–mediated proteasome recovery: Upon PI exposure, cells activate Nrf1, which is proteolytically processed by DDI2. Processed Nrf1 translocates to the nucleus and upregulates proteasome assembly chaperone 1 along with multiple 26S subunits, actively promoting proteasome reassembly to counteract drug effects. (5) XBP1 mutations: Mutations in XBP1 and the subsequent downregulation of its signaling pathway partially impede plasma cell differentiation and reduce baseline ER stress, shielding MM cells from PI‐induced proteotoxic collapse. (6) Protective SUMOylation: SUMOylation serves as an intrinsic defense mechanism, conferring direct cellular protection under conditions of therapeutic DNA damage and proteotoxic stress. (7) RPS3‐mediated protumor pathway activation: RPS3 acts as a critical determinant of drug resistance; it interacts with TRIP13 to constitutively activate the pro‐survival NF‐κB signaling pathway and bypass proteasomal blockades. (8) Overexpression of ABC transporters that enhance drug efflux: Upregulation of ABC transporters actively pump PIs out of the cell. This process markedly reduces intracellular drug exposure and prevents the initiation of programmed cell death. The figure was created by the authors using Figdraw (https://www.figdraw.com/) and does not reproduce or adapt any previously published material.

Abbreviations: PIs (Proteasome Inhibitors), proteasome 20S subunit β5 (PSMB5), ER (Endoplasmic Reticulum), UPR (Unfolded Protein Response), BMME (Bone Marrow Microenvironment), MM (Multiple Myeloma), PSMB5 (Proteasome 20S Subunit β5), PSMA3 (Proteasome 20S Subunit α3), CAM‐DR (Cell‐Adhesion Mediated Drug Resistance), BH4 (Tetrahydrobiopterin), DUBs (Deubiquitinating Enzymes), Sec. 61 (Sec. 61 Translocon Complex), DDI2 (DNA‐Damage Inducible 1 Homolog 2), Nrf1 (NF‐E2‐Related Factor 1), XBP1 (X‐box Binding Protein 1), SUMOylation (Small Ubiquitin‐Like Modifier Modification), RPS3 (Ribosomal Protein S3), NF‐κB (Nuclear Factor‐κB), ABC (ATP‐Binding Cassette), IRE1α (Inositol‐requiring enzyme 1 α), TRIP13 (Thyroid Hormone Receptor Interactor 13), P‐gp (P‐glycoprotein).

### 2.3 mAbs

In the field of immunotherapy for MM, targeted mAbs have become one of the key therapeutic modalities, and the development of mAbs targeting plasma cell surface antigens such as CD38, signaling lymphocytic activation molecule family member 7 (SLAMF7), and B cell maturation antigen (BCMA) has progressively matured. These agents exert anti‐myeloma activity through antibody‐dependent cell‐mediated cytotoxicity (ADCC), complement‐dependent cytotoxicity (CDC), antibody‐dependent cellular phagocytosis (ADCP), and broader immunomodulatory effects. In addition, inhibition of ectoenzyme activity and direct induction of apoptosis may further contribute to the capacity of these antibodies to eliminate MM cells [57].

One major mechanism of acquired resistance to ADCC is downregulation of target antigen expression [57, 58]. During anti‐CD38 therapy, reductions in CD38 expression have been observed and are thought to reflect clonal selection. In addition, trogocytosis and microvesicle shedding have been implicated, leading to transfer of CD38‐antibody complexes away from MM cells [59]. Previous studies have shown that binding of all‐trans retinoic acid (ATRA) to retinoic acid receptors modulates the gene expression of surface receptors, leading to increased CD38 expression and reduced expression of CD55 and CD59 in MM cell lines and primary MM cells [58]; supplementation with ATRA can reverse this effect [60]. In parallel, BMSCs exert protective effects that further dampen ADCC and promote resistance to daratumumab, while YM155 can partially abrogate this stromal protection [61].

One of the major mechanisms underlying resistance to CDC is the overexpression of CD55 and CD59, which suppresses activation of the complement system. Pharmacological inhibition of membrane complements regulators, including CD46 and CD59, has been shown to enhance daratumumab‐ and isatuximab‐mediated CDC in MM models [62]. For classical IgG antibodies, the formation of ordered hexamers on the cell membrane is a critical step for efficient C1q recruitment and initiation of CDC. Insufficient hexamerization capacity of therapeutic antibodies represents another mechanism of resistance within the CDC pathway [63, 64]. Studies have shown that HexaBody‐CD38 remains effective even in cells with low CD38 expression [65]. Moreover, within the BMME, soluble CD38 and extracellular vesicles carrying CD38‐antibody complexes can act as decoys. High expression of complement inhibitory factors can further consume or neutralize complement activity, thereby attenuating CDC [66].

**Figure 3:**
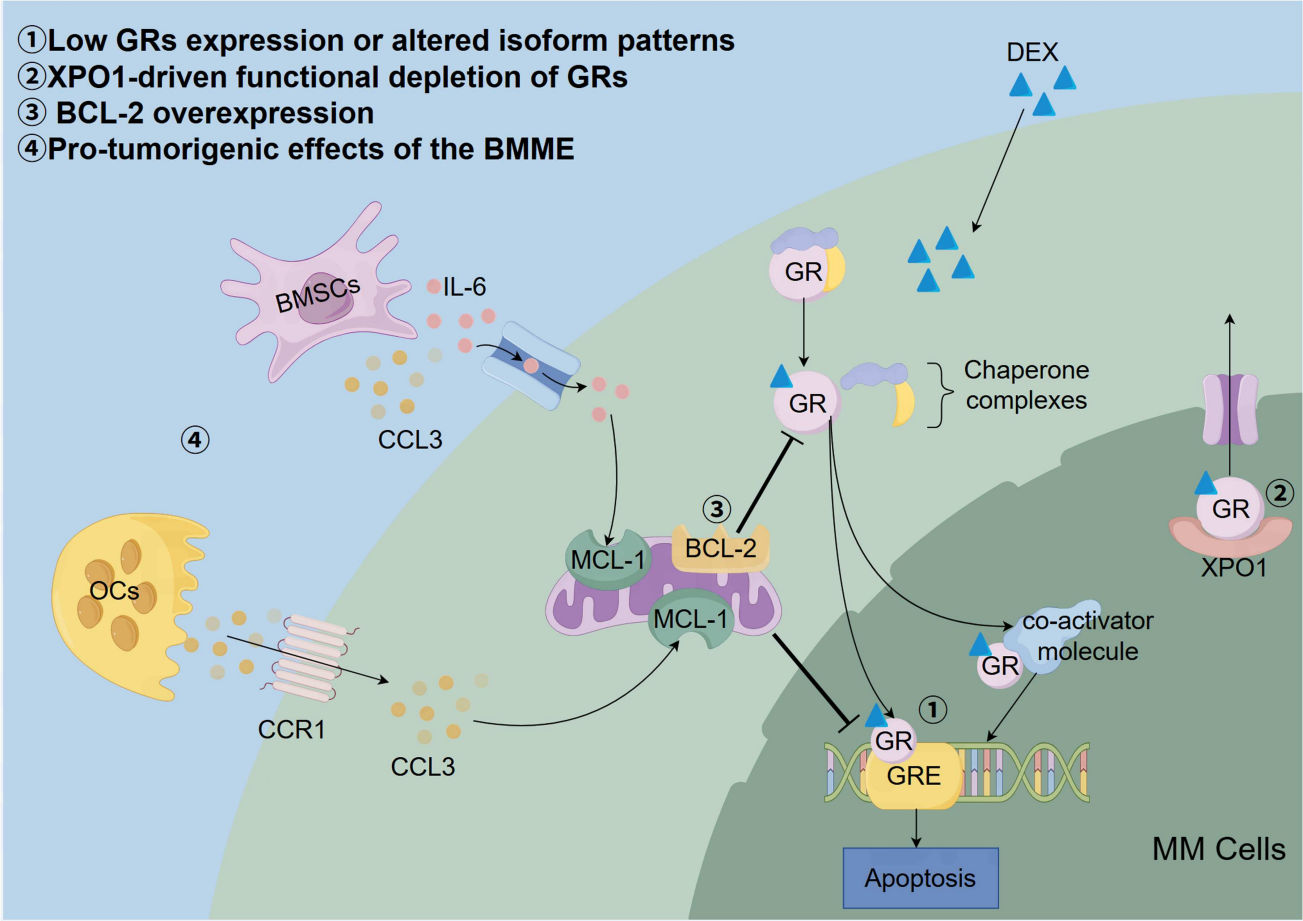
Mechanisms of mAbs action and resistance in MM.

The CD47‐SIRPα axis constitutes an anti‐phagocytic barrier in MM, and CD47 blockade enhances macrophage‐mediated phagocytosis of MM cells. Blockade of the CD47 checkpoint enhances the cooperative phagocytosis of tumor‐targeting antibodies [67]. The therapeutic efficacy of elotuzumab relies predominantly on ADCC mediated by NK cells and ADCP mediated by macrophages. When NK cell function is suppressed within the BMME, resistance can still emerge even if MM cells continue to express SLAMF7 [68] (Figure 3).

Targeting the BMME to overcome resistance to mAbs has become a major focus of current research. Counteracting target antigen shedding or downregulation is critical in MM, and rotating therapeutic targets may delay or bypass resistance development. Inhibition of the CD39/CD73 adenosine axis has been shown to potentiate antitumor immune responses. Pharmacologic modulation of adenosine signaling may represent a promising therapeutic avenue [69].

Canonical Action: Therapeutic mAbs eliminate MM cells expressing surface antigens through ADCC, CDC, and ADCP. These processes are supported by broader immunomodulatory effects, ectoenzyme inhibition, and the direct induction of cell apoptosis.

Mechanisms of Resistance: (1) Decreased target antigen expression and shedding: Clonal selection drives the progressive downregulation of target antigens on the MM cell surface. Furthermore, trogocytosis and microvesicle shedding physically strip and transfer CD38‐antibody complexes away from the tumor cells, preventing effective antibody engagement and subsequent ADCC. (2) Suppression of complement system activation: The marked overexpression of membrane‐bound complement regulatory proteins, specifically CD55 and CD59, on MM cells directly suppresses the cascade activation of the complement system, blocking the downstream assembly of the membrane attack complex. (3) Insufficient antibody hexamerization and complement sink effects: The failure of classical IgG therapeutic antibodies to form ordered hexamers on the target cell membrane compromises efficient C1q recruitment, thereby halting the initiation of CDC. Concurrently, soluble target antigens and circulating extracellular vesicles carrying antigen‐antibody complexes act as decoy “complement sinks” that consume and neutralize complement activity away from the actual tumor cell surface. (4) Impaired effector cell function within the BMME: Immunosuppressive cues within the BMME paralyze host effector cells; for instance, the suppression of NK cell and macrophage fitness disables ADCC and ADCP, while the tumor‐intrinsic upregulation of CD47 delivers a strong inhibitory signal to macrophages. The figure was created by the authors using Figdraw (https://www.figdraw.com/) and does not reproduce or adapt any previously published material.

Abbreviations: MM (Multiple Myeloma), mAbs (Monoclonal Antibodies), ADCC (Antibody‐Dependent Cell‐Mediated Cytotoxicity), CDC (Complement‐Dependent Cytotoxicity), BMME (Bone Marrow Microenvironment), ADCP (Antibody‐Dependent Cellular Phagocytosis), NK (Natural Killer), NEU (Neutrophil), MAC (Membrane Attack Complex), SLAMF7 (Signaling Lymphocytic Activation Molecule Family Member 7), EAT‐2 (Ewing's Sarcoma‐associated Transcript 2), SIRPα (Signal Regulatory Protein α).

### 2.4 GCs

Despite many novel MM therapies, glucocorticoids remain first‐line staples, especially in older or comorbid patients in whom low‐dose dexamethasone combinations are widely used [70]. Dexamethasone (DEX) exerts its principal therapeutic effect by binding to glucocorticoid receptors (GRs), promoting their translocation into the nucleus and activating glucocorticoid response element (GRE)‐dependent transcription, which in turn activates the intrinsic apoptotic pathway [71].

The development of resistance to GCs is associated with an overall reduction in GRs levels and altered expression patterns of GRs isoforms. In GC‐sensitive MM cell lines, GR‐α predominates and declines with stepwise resistance, while late‐stage resistant lines may shift toward dominant GR‐β [72]. Exportin 1 (XPO1)‐mediated nuclear export functionally depletes glucocorticoid receptors by driving cytoplasmic sequestration of activated GRs and tumor suppressor proteins, thereby blunting GCs‐induced transcription. This mechanism contributes to dexamethasone insensitivity in RRMM and provides a biological rationale for XPO1 inhibitors such as selinexor combined with low‐dose DEX, supporting clinical development and regulatory approval [73, 74, 75]. In addition, covalent binding of selinexor to XPO1 promotes degradation of XPO1 itself via the ubiquitin‐proteasome pathway, resulting in more sustained inhibition of nuclear export [73, 74].

**Figure 4:**
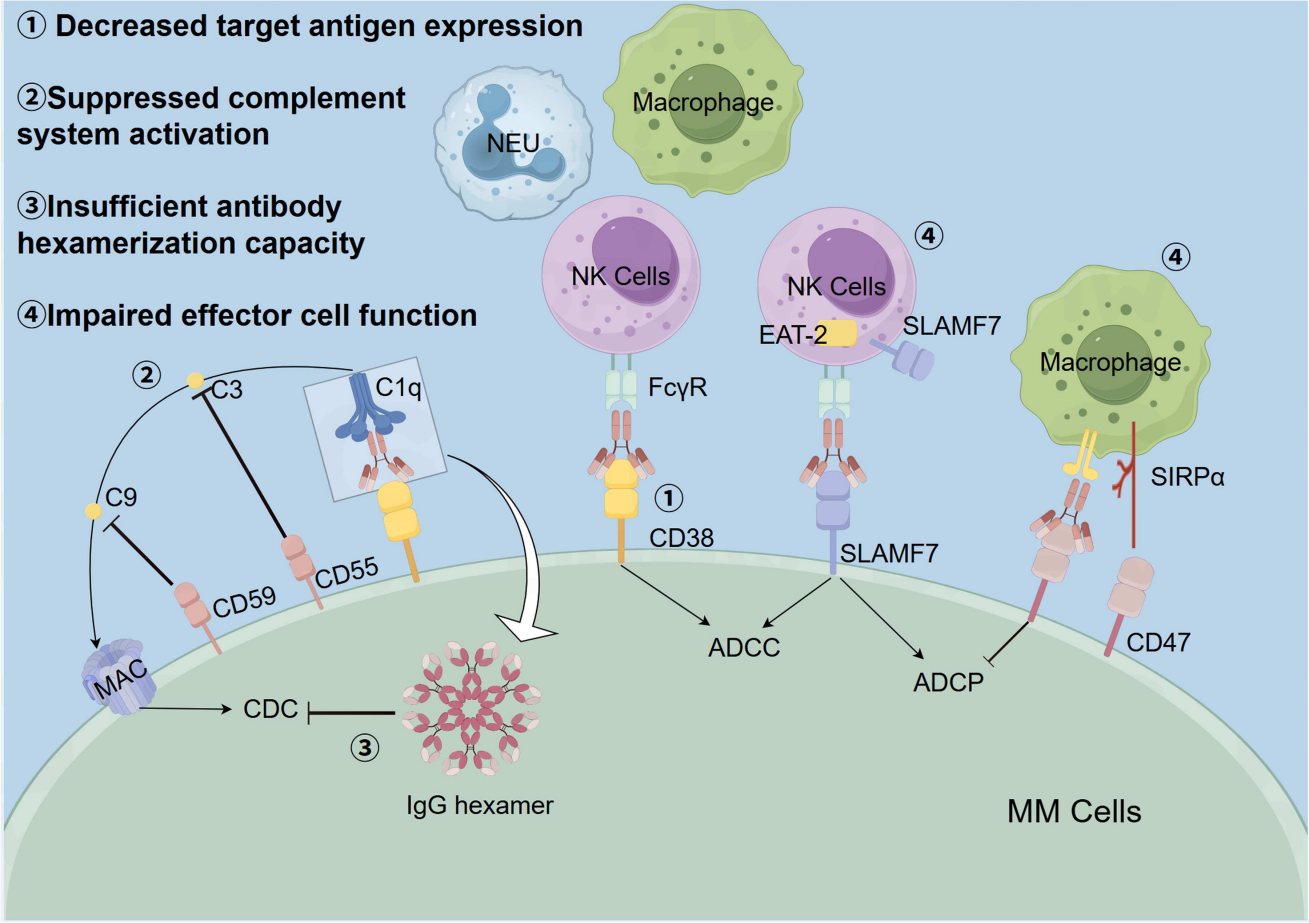
Mechanisms of GCs action and resistance in MM.

Several studies have demonstrated that the antiapoptotic proteins MCL‐1 and BCL‐2 play critical roles in MM cell survival and DR (Figure 4). In preclinical studies, combining MCL‐1 inhibitors with DEX has shown potential to reverse resistance, particularly in the context of MCL‐1 upregulation [76]. In addition, the BCL‐2 inhibitor venetoclax, when combined with DEX, has achieved deep responses in specific subgroups such as patients harboring t(11;14) translocation. Dual targeting of MCL‐1 and BCL‐2 therefore represents a promising strategy to overcome resistance [77]. Studies have shown that C‐C chemokine receptor 1 (CCR1) activation attenuates DEX‐induced apoptosis and thereby promotes the development of resistance [78]. This BMME‐mediated alternative survival pathway underscores the profound influence of the BMME on tumor cell biology (Table 2).

**Table 2:** Key drug resistance mechanisms and therapeutic strategies.

Drug class	Potential mechanisms of drug resistance	Counteracting approaches to overcome drug resistance
Immunomodulatory drugs	1. Aberrations in the cereblon axis: Downregulation of cereblon expression caused by cereblon mutations, copy‐number loss, or enhancer methylation [34, 35]. 2. Transcriptional and pathway reprogramming [36, 37, 38]. 3. Microenvironment‐mediated bypass signaling sustains cell survival and contributes to resistance [40, 41].	1. Introducing synthetic lethal combinations to reinforce the cereblon‐dependent ubiquitination axis [36, 37]. 2. Combining pathway inhibitors to block super‐enhancer‐mediated oncogenic transcription [38, 39]. 3. Blocking microenvironment‐derived cytokine bypass signaling [40, 41]. 4. Implementing temporally optimized combination therapies targeting proteostasis or stress‐response pathways [49, 50].
Proteasome inhibitors	1. Mutations at proteasome drug‐binding sites and compensatory proteasome reassembly [9, 45, 51]. 2. Restoration of stress adaptation and detoxification capacity: activation of deubiquitinating enzymes, increased endoplasmic reticulum‐associated trafficking, and attenuation of endoplasmic reticulum stress [48, 49, 50, 52]. 3. Enhanced intracellular buffering capacity and increased drug efflux [53, 54, 56].	1. Selecting next‐generation proteasome inhibitors based on mutational profiles [9]. 2. Blocking nascent polypeptide trafficking with targeted inhibitors to induce terminal endoplasmic reticulum stress [49, 50]. 3. Targeting ATP‐binding cassette transporter‐mediated drug efflux to increase intracellular drug exposure [56].
Monoclonal antibodies	1. Target antigen loss or depletion: clonal selection or trogocytosis; soluble antigens or extracellular vesicles acting as complement or antibody decoy sinks [57, 58, 59, 66]. 2. Immune escape and biophysical barriers: overexpression of immune‐evasive surface markers and insufficient intrinsic hexamerization capacity of therapeutic antibodies [58, 63, 64, 65, 67]. 3. Microenvironment‐driven immune exhaustion: suppressive cellular components within the bone marrow microenvironment paralyze NK cell and T cell function [61, 68, 69].	1. Supplementing with all‐trans retinoic acid to enhance CD38 expression and downregulate CD55/CD59 [58, 60]. 2. Overcoming monoclonal antibody resistance by inhibiting complement regulatory proteins or by applying next‐generation antibodies with superior hexamerization capacity [62, 65]. 3. Blocking the CD47 checkpoint to enhance macrophage‐mediated cooperative phagocytosis [67]. 4. Rotating therapeutic targets to potentially delay or bypass antigen‐specific resistance [57, 58].
Glucocorticoids	1. Functional impairment of the glucocorticoid receptor [71, 72]. 2. Blocked nuclear translocation: excessive Exportin 1 activity mediates nuclear export and cytoplasmic sequestration of activated glucocorticoid receptor [73, 74, 75]. 3. Overexpression of anti‐apoptotic proteins and activation of survival bypass pathways, thereby attenuating the pro‐apoptotic effects of glucocorticoids [76, 77, 78].	1. Combining Exportin 1 inhibitors to prevent glucocorticoid receptor nuclear export and maintain the intranuclear levels of tumor‐suppressive proteins [73, 74, 75]. 2. Combining inhibitors of anti‐apoptotic proteins to trigger intrinsic apoptosis [76, 77].

Canonical Action: DEX binds to cytoplasmic GRs, promoting their homodimerization and subsequent translocation into the nucleus. Once in the nucleus, activated GRs bind to specific GREs to modulate gene transcription, thereby triggering the intrinsic apoptotic pathway.

Mechanisms of Resistance: (1) Reduced GR expression or altered isoform patterns: Stepwise resistance to GCs is driven by an overall reduction in total GR levels or a profound shift in expression patterns among GR isoforms. In sensitive cells, the dominant GR‐α isoform mediates drug responsiveness; however, resistant cells exhibit a significant decline in GR‐α coupled with a phenotypic shift toward the dominant, ligand‐insensitive GR‐β isoform, blunting receptor‐level activation. (2) XPO1‐driven functional depletion of nuclear GRs: Hyperactive XPO1 actively mediates the nuclear export of activated GRs and crucial tumor suppressor proteins, forcing their cytoplasmic sequestration. This nuclear transport defect functionally depletes the pool of active, nuclear‐localized GRs, thereby directly preventing GREs‐dependent transcription and causing profound dexamethasone insensitivity in relapsed or refractory disease. (3) Overexpression of antiapoptotic proteins: The systematic upregulation of core antiapoptotic proteins, specifically MCL‐1 and BCL‐2, directly blocks downstream death execution. These proteins elevate the apoptotic threshold and sustain MM cell survival, creating distinct therapeutic vulnerabilities that require targeted intervention. (4) Pro‐tumorigenic cues and alternative survival pathways from the BMME: Environmental factors within the BMME actively counteract GC‐induced death. For instance, the activation of CCR1 by niche signals establishes a robust alternative survival branch that directly attenuates DEX‐induced apoptosis, demonstrating the profound protective impact of the microenvironmental niche on tumor biology. The figure was created by the authors using Figdraw (https://www.figdraw.com/) and does not reproduce or adapt any previously published material.

Abbreviations: GCs (Glucocorticoids), DEX (Dexamethasone), GR‐α (Glucocorticoid Receptor α), GR‐β (Glucocorticoid Receptor β), GRs (Glucocorticoid Receptors), GREs (Glucocorticoid Response Elements), XPO1 (Exportin 1), MCL‐1 (Myeloid Cell Leukemia 1), BCL‐2 (B‐Cell Lymphoma 2), OCs (Osteoclasts), CCL3 (C‐C chemokine ligand 3), MM (Multiple Myeloma), BMME (Bone Marrow Microenvironment), CCR1 (C‐C chemokine receptor 1).

## 3 Environment‐Mediated Drug Resistance in Multiple Myeloma

### 3.1 Cell Adhesion–Mediated Drug Resistance (CAM‐DR)

Bone marrow endothelial cells (BMECs) and BMSCs provide pro‐proliferative niches characterized by the expression of endothelial lineage markers and activation of multiple adhesion and angiogenic pathways, thereby supporting MM cell survival and treatment resistance [79]. Among the adhesion molecules expressed on MM cells, very late antigen 4 (VLA‐4) and its ligand vascular cell adhesion molecule 1 (VCAM‐1) constitute a central adhesion axis that underlies the retention and proliferative advantage of MM cells within the bone marrow niche [80]. Genetic evidence further shows that deletion of the α4 subunit in MM cells markedly reduces bone marrow engraftment, increases extramedullary dissemination, and prolongs survival in murine models [81]. VLA‐4‐targeted radiotracers can sensitively detect tumor burden and assess BTZ response in MM mouse models [82], and show favorable safety and biodistribution profiles in patients [83]. VLA‐4‐targeted nanoparticles can exploit the elevated VLA‐4 expression of drug‐resistant MM cells to selectively deliver therapeutic payloads and improve antitumor efficacy in preclinical models [84].

The C‐X‐C motif chemokine ligand 12 (CXCL12)/C‐X‐C chemokine receptor type 4 (CXCR4) axis is one of the principal drivers of MM cell trafficking to and retention within protective bone marrow niches [85]. This signaling not only activates survival pathways but also establishes a positive feedback loop involving CXCL12, thereby increasing resistance to PIs and other agents [85]. Moreover, the CXCL12/CXCR4 axis also mediates intercellular mitochondrial transfer from BMSCs to MM cells, thereby reprogramming tumor energy metabolism and reinforcing an antiapoptotic phenotype [86]. Short‐term pharmacologic inhibition of CXCR4 with agents such as plerixafor or ulocuplumab can mobilize MM cells out of their protective niches; this concept has been supported by recent clinical and translational studies [87, 88].

BMSC‐derived hyaluronic acid (HA) promotes homophilic aggregation of CD44 variant‐expressing MM cells under shear stress and contributes to the development of extramedullary disease [89]. CD44 downregulation disrupts actin cytoskeletal organization and reduces the migration and adhesion of MM cells [90]. The key HA network linker protein can further broaden cross‐drug tolerance, as either exogenous supplementation or high stromal expression can enhance NF‐κB activation and protect MM cells from treatment‐induced cell death [91].

Tunneling nanotubes (TNTs) within the BMME mediate mitochondrial transfer between BMSCs and MM cells [92, 93]. Functional studies using primary patient samples have confirmed that, under chemotherapeutic stress, MM cells acquire increased mitochondrial content from autologous BMSCs, accompanied by elevated ATP levels, reduced mitochondrial superoxide levels, and consequently enhanced drug tolerance [94]. Under hypoxic or glucose‐deprived stress, MM cells export damaged mitochondria to BMSCs via TNTs, where they are processed through transmitophagy and TNTs‐mediated relay transport [92]. TNTs depend on the actin cytoskeleton and are functionally linked to CD38; inhibition of actin polymerization or interference with CD38 reduces mitochondrial transfer and the associated metabolic advantages [93].

**Figure 5:**
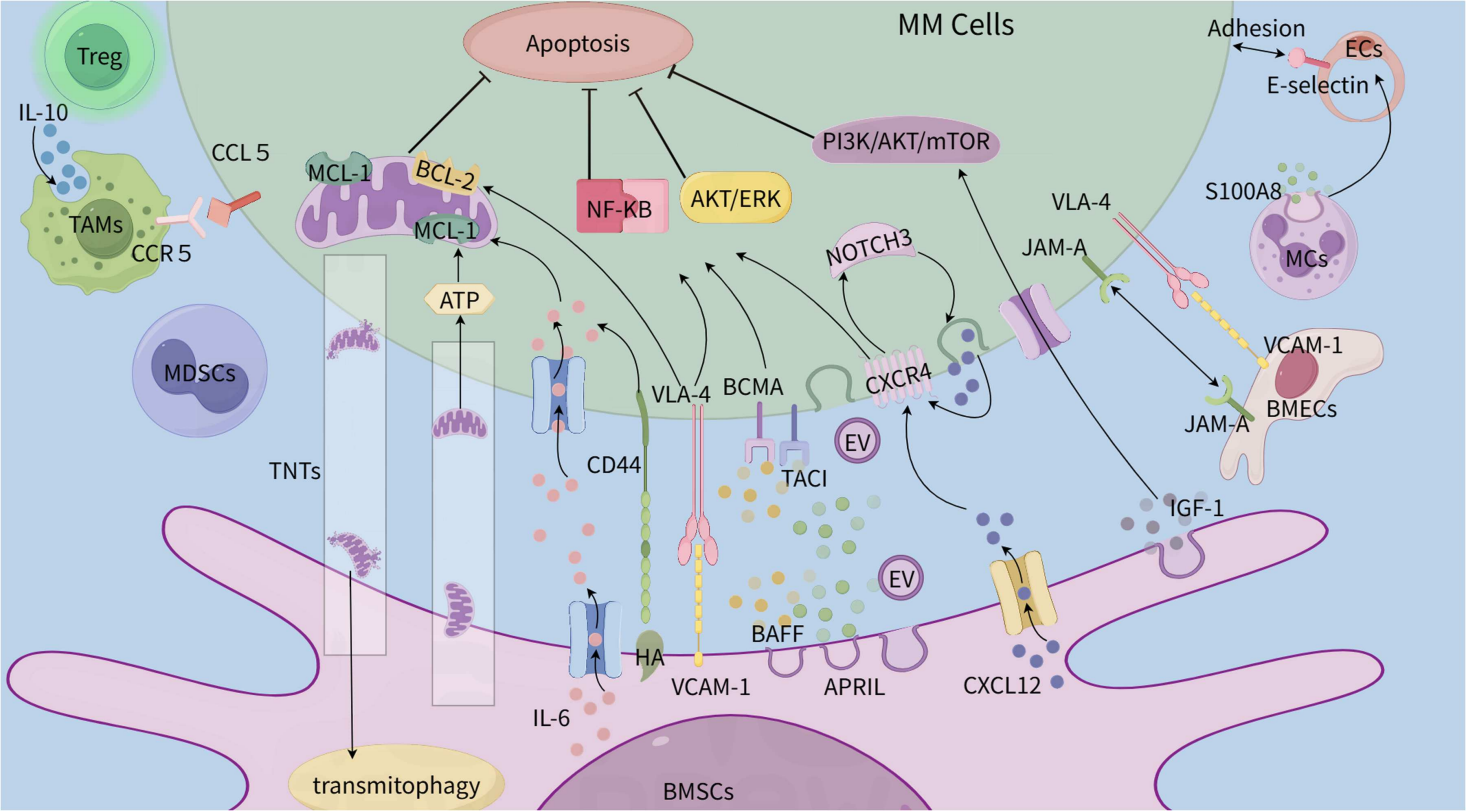
BMME‐driven CAM‐DR and SFM‐DR in MM.

Beyond these landmark findings, additional resistance mechanisms continue to emerge. E‐selectin–dependent vascular adhesion niches have been shown to enhance tolerance to PIs, and E‐selectin–targeted lipid nanoparticles improve the antitumor efficacy of BTZ while reducing toxicity [95]. JAM‐A (junctional adhesion molecule A) forms a proangiogenic adhesion circuit between MM cells and BMECs, and blockade of JAM‐A disrupts this circuit and restores treatment responsiveness [96] (Figure 5). A highly sialylated glycan code on the surface of MM cells promotes endothelial adhesion and drives CAM‐DR [97]. These findings provide a rationale for evaluating treatment sequences in which transient niche disruption precedes or accompanies systemic anti‐MM therapy. However, whether this strategy prevents residual tumor cells from re‐establishing protective niche interactions requires further clinical validation [87].

### 3.2 Soluble Factor–Mediated Drug Resistance (SFM‐DR)

IL‐6 signals through janus kinase (JAK)/STAT3 to upregulate MCL‐1 and rapidly raises the apoptotic threshold, thereby directly inducing SFM‐DR to PIs and IMiDs [98]. While a Dickkopf‐1‐driven autocrine IL‐6 loop further enhances BTZ tolerance via noncanonical NF‐κB signaling and induces CD138 downregulation and clonal escape [99]. Insulin‐like growth factor 1 receptor (IGF‐1R) mutations can alter receptor phosphorylation or downstream signaling in a mutation‐ and cellular‐context‐dependent manner, although their effects on MM‐cell viability and treatment response remain incompletely defined [100]. Multiomic profiling and dependency analyses have identified insulin receptor substrate 1 as a critical relay in Insulin‐like growth factor 1 (IGF‐1) signaling [101].

B‐cell activating factor (BAFF) is secreted by cells within the BMME and binds to receptors on MM cells, predominantly transmembrane activator and CAML interactor (TACI) and, to a lesser extent, BCMA. This interaction rapidly activates survival pathways, and is considered one of the fundamental drivers of SFM‐DR [102]. A proliferation‐inducing ligand (APRIL) binds BCMA and TACI and promotes MM‐cell growth, survival, adhesion, and immunosuppressive interactions within the bone marrow niche [103]. Through TACI‐mediated signaling, APRIL also promotes regulatory T‐cell activation, reinforces immunosuppression within the bone marrow niche, and indirectly protects MM cells from treatment‐induced cytotoxicity [104].

EVs, particularly exosomes, are key mediators of intercellular communication and the development of DR in MM. EVs‐mediated exchange between MM cells and BMSCs transfers noncoding RNAs, proteins, metabolic enzymes, and mitochondrial components, thereby reshaping the metabolic and signaling networks of recipient cells and promoting their survival and resistance [105]. EVs released by MM cells are enriched for miR‐21 and miR‐146a, which can reprogram BMSCs and further support tumor cell survival and DR [106].

Beyond the landmark discoveries described above, several additional BMME pathways have been implicated in the development of resistance. CD39 and CD73, expressed on MM cells and bone marrow dendritic cells, hydrolyze ATP to adenosine, which activates A2A receptors, suppresses antitumor immunity, and reduces drug‐mediated cytotoxicity [69, 107]. The CD73 inhibitor ORIC‐533 lowers adenosine levels and enhances T‐cell–mediated lysis in patient‐derived coculture systems, and in combination with daratumumab markedly amplifies anti‐myeloma activity [107]. The soluble myeloid‐derived proteins S100A8/A9 promote the expansion of megakaryocytes that support MM progression [108]. IL‐10 signaling sustains immunosuppression and DR in TAMs. In MM models, blockade of the IL‐10 receptor can reprogram TAMs and reverse drug insensitivity [109]. The CCR5 antagonist maraviroc suppresses M2 polarization and cooperates with BTZ to enhance cytotoxicity, supporting the CCL5/CCR5 axis as a complementary therapeutic target for further clinical translation [110] (Figure 5).

CAM‐DR: Direct cell‐to‐cell and cell‐to‐matrix interactions within the niche activate pro‐proliferative and pro‐survival pathways. The canonical VLA‐4/VCAM‐1 adhesion axis provides MM cells with retention and proliferative advantages, while the CXCL12/CXCR4 pathway drives bone marrow homing and forms a self‐reinforcing survival loop. Additional structural components—including HA/CD44 interactions that trigger NF‐κB activation, E‐selectin vascular niches, JAM‐A proangiogenic circuits, and sialylated glycan codes—broaden multidrug tolerance. Crucially, intercellular mitochondrial transfer mediated by TNTs and the CXCR4 axis infuses MM cells with stromal‐derived mitochondria, elevating ATP levels and reducing oxidative stress to reinforce a highly drug‐tolerant, anti‐apoptotic phenotype.

SFM‐DR: Soluble mediators and EVs build an immunosuppressive, drug‐resistant sanctuary. BMSCs‐derived IL‐6 signals via the JAK/STAT3 axis to directly upregulate MCL‐1 and rapidly raise the apoptotic threshold, while alternative autocrine loops trigger noncanonical NF‐κB activation for clonal escape. Gains in IGF‐1/IGF‐1R signaling and stromal BAFF/APRIL secretion further fuel survival cascades, activate regulatory T‐cells, and competitively impede BCMA‐directed therapies. Concurrently, EV‐borne cargoes transfer molecular messages to reshape metabolic profiles and enhance proteotoxic stress buffering. Finally, the CD39/CD73‐adenosine immunosuppressive axis, S100A8/A9 proteins, macrophage‐derived IL‐10, and CCL5/CCR5 signaling collectively paralyze host antitumor immunity. The figure was created by the authors using Figdraw (https://www.figdraw.com/) and does not reproduce or adapt any previously published material.

Abbreviations: CAM‐DR (Cell‐Adhesion Mediated Drug Resistance), SFM‐DR (Soluble Factor–Mediated Drug Resistance), VLA‐4 (Very Late Antigen 4), VCAM‐1 (Vascular Cell Adhesion Molecule 1), CXCL12 (C‐X‐C motif chemokine ligand 12), CXCR4 (C‐X‐C chemokine receptor type 4), HA (Hyaluronic Acid), NF‐κB (Nuclear Factor‐κB), E‐selectin (Endothelial Selectin), JAM‐A (Junctional Adhesion Molecule A), TNTs (Tunneling Nanotubes), BMSCs (Bone Marrow Stromal Cells), IL‐6 (Interleukin‐6), JAK (Janus Kinase), STAT3 (Signal Transducer and Activator of Transcription 3), MCL‐1(Myeloid Cell Leukemia 1), IGF‐1 (Insulin‐Like Growth Factor‐1), IGF‐1R (Insulin‐Like Growth Factor 1 Receptor), BAFF (B‐Cell Activating Factor), APRIL (A Proliferation‐Inducing Ligand), BCMA (B Cell Maturation Antigen), TACI (Transmembrane Activator and CAML Interactor), EVs (Extracellular Vesicles), CCL5 (C‐C Chemokine Ligand 5), CCR5 (C‐C Chemokine Receptor 5), NOTCH3 (Neurogenic Locus Notch Homolog Protein 3), Treg (Regulatory T Cell), MDSCs (Myeloid‐Derived Suppressor Cells), BMECs (Bone Marrow Endothelial Cells), ECs (Endothelial Cells), MCs (Myeloid Cells), PI3K (Phosphoinositide 3‐Kinase), ERK (Extracellular Signal‐Regulated Kinase).

## 4 Conclusion

In summary, drug resistance in MM is no longer regarded as a single, isolated molecular lesion, but rather as a dynamic and multidimensional ecosystem shaped by continuous reciprocal evolution between tumor‐intrinsic cellular pathway rewiring and the BMME. To address the limited forward‐looking perspective of previous conclusions, this review proposes that future efforts to overcome MM drug resistance and achieve clinically meaningful breakthroughs will center on four major frontier directions.

First, integrated proteogenomic analyses can identify genetic subtype‐specific signaling alterations, prognostic protein signatures, and potential therapeutic vulnerabilities that are not fully captured by genomic or transcriptomic profiling alone [101]. Second, longitudinal genomic analyses have shown that biallelic inactivation of BCMA or GPRC5D can emerge after exposure to the corresponding CAR‐T‐cells or T‐cell engagers and may be undetectable at treatment initiation [111]. These findings support dynamic surveillance of target‐antigen alterations during therapy rather than reliance solely on baseline mutational assessment. Meanwhile, the development of next‐generation multi‐target CAR‐T architectures, such as bispecific BCMA/B7‐H3 CAR‐T‐cells, holds considerable promise for preventing single‐antigen escape and achieving more complete eradication of heterogeneous tumor clones [112]. Third, interventions targeting cellular communication within the bone marrow microenvironment have advanced to the level of fine‐scale biophysical structures and local immunoregulatory targets. These include blockade of TNTs‐mediated stromal‐to‐tumor mitochondrial transfer [92], which may dismantle the bioenergetic shield supporting MM cells, as well as precision relief of microenvironmental immune suppression through targets such as the CD73 ectonucleotidase [107] and the IL‐10R‐dependent macrophage remodeling pathway [109]. Such strategies represent highly promising approaches to reversing extrinsic cell adhesion‐mediated drug resistance and soluble factor‐mediated drug resistance. Finally, high‐throughput ex vivo drug response profiling of primary patient samples [113], when integrated with individualized resistance‐network mapping, is driving anti‐MM therapy away from empirical drug stacking and toward truly mechanism‐guided precision treatment. With the convergent evolution of these multidimensional strategies, MM may ultimately be transformed from a malignancy characterized by frequent relapse and eventual pan‐refractoriness into a controllable disease in which long‐term progression‐free survival, or even functional cure, becomes achievable.

## Author Contributions

The review was conceptualized by Xiaotao Wang. The original draft was prepared by Yixuan Chen. Figures were designed and prepared by Wenming Huang and Yixuan Chen. The manuscript was reviewed and edited by Wenming Huang and Mingxuan Tang. The work was supervised by Xiaotao Wang. All authors have read and agreed to the published version of the manuscript.

## Conflicts of Interest

The authors declare no conflicts of interest.

## Data Availability

Data sharing is not applicable to this article, as no new data were created or analyzed in this study.
